# Supplementary material for: Comparison of Quality Changes in Eurasian Perch (Perca fluviatilis L.) Fillets Originated from Two Different Rearing Systems during Frozen and Refrigerated Storage
Source: Foods. 2021 Jun 17;10(6):1405. doi: 10.3390/foods10061405 (PMC8233806; doi:10.3390/foods10061405)
Supplement: Supplementary file 1 [file foods-10-01405-s001.zip › foods-1242752-supplementary.pdf]

Table S1. Protein identification by PMF (Peptide mass fingerprinting) and MALDI TOF/TOF.

Protein Spot.	Protein Identified	Accession Number	Function	Mascot Score	Expectation	Peptides in Match	Mass (kDa)	Isoelectric Point
1	Actin	P68140	Skeletal muscle protein	127	1.1×10^{-9}	11	43	5.22
2	GAPDH	Q5XJ10	Carbohydrate degradation; glycolysis	68	0.00095	8	37	8.20
3	LDH A-chain	O93543	Pyruvate fermentation to lactate	58	0.009	7	37	6.35
4	Beta-enolase	B5DGQ7	Carbohydrate degradation; glycolysis; pyruvate from D-glyceraldehyde 3-phosphate	63	0.0027	8	49	6.61
5	Triosephosphate isomerase	Q90XG0	Glycolysis Gluconeogenesis	79	7.2×10^{-5}	8	28	6.45

According to the protein function from Peptide Mass Fingerprinting online Mascot server, the identified proteins were actin, GAPDH, LDH A-chain, Beta-enolase, Triose phosphate isomerase and Beta-enolase. GAPDH, Glyceraldehyde 3-phosphate dehydrogenase. LDH A-chain, Lactate dehydrogenase A-chain.
